# Impact of the COVID-19 Pandemic on Participation in Community-Dwelling Older Adults: A Cross-Sectional Analysis

**DOI:** 10.1093/geroni/igab046.2703

**Published:** 2021-12-17

**Authors:** Khang Nguyen, Luciana Macedo, Brenda Vrkljan, Renata Kirkwood, Jinhui Ma, Marla Beauchamp

**Affiliations:** 1 McMaster University, Hamilton, Ontario, Canada; 2 McMaster University, McMaster University, Ontario, Canada

## Abstract

Public health guidelines to prevent spreading COVID-19 place older adults at risk of loneliness and social isolation. Evidence suggests that participation protects older adults from such detrimental outcomes, therefore we aimed to identify the factors associated with participation in life roles among older adults living in the community during the COVID-19 pandemic. We conducted a telesurvey on a random sample of community-dwelling older adults living in Hamilton, Ontario, Canada, between May and July 2020. Outcome measures included participation in life roles, physical function, physical activity, mobility, mental health, nutrition, and demographics. We conducted two multivariate regression analyses with the Late Life Disability Instrument’s (LLDI) frequency and limitations scales as the dependent variables. Candidate factors were organized by International Classification of Functioning, Disability, and Health (ICF) framework domains; personal factors, body functions and structures, activities, and environmental factors. A total of 272 older adults completed the telesurvey (mean age 78 ±7.3 yrs, 70% female). Age, using walking aids, driving status, household income, education, mental health, nutrition, physical function, and dwelling type explained 47.1% (p<0.001) of the variance observed in LLDI frequency scores. Using walking aids, driving status, receiving health assistance, mental health, and physical function explained 33.9% (p<0.001) of the variance observed in LLDI limitation scores. These findings highlight factors from multiple ICF domains that are associated with participation limitation and frequency among older adults during the pandemic. Our findings have implications for developing public health initiatives to mitigate the effects of the pandemic on the participation of older adults.

